# Genetic structure of four plasmids found in *Acinetobacter baumannii* isolate D36 belonging to lineage 2 of global clone 1

**DOI:** 10.1371/journal.pone.0204357

**Published:** 2018-09-27

**Authors:** Mohammad Hamidian, Ruth M. Hall

**Affiliations:** 1 The ithree institute, University of Technology Sydney, Ultimo, New South Wales, Australia; 2 School of Life and Environmental Sciences, The University of Sydney, New South Wales, Australia; University of Manchester, UNITED KINGDOM

## Abstract

Four plasmids ranging in size from 4.7 to 44.7 kb found in the extensively antibiotic resistant *Acinetobacter baumannii* isolate D36 that belongs to lineage 2 of global clone 1 were examined. D36 includes two cryptic plasmids and two carrying antibiotic resistance genes. The smallest plasmid pD36-1 (4.7 kb) carries no resistance genes but includes *mobA* and *mobC* mobilisation genes related to those found in pRAY* (pD36-2, 6,078 bp) that also carries the *aadB* gentamicin, kanamycin and tobramycin resistance gene cassette. These two plasmids do not encode a Rep protein. Plasmid pRAY* was found to be mobilised at high frequency by the large conjugative plasmid pA297-3 but a pRAY* derivative lacking the *mobA* and *mobC* genes was not. The two larger plasmids, pD36-3 and pD36-4, encode Rep_3 family proteins (Pfam1051). The cryptic plasmid pD36-3 (6.2 kb) has RepAci1 and pD36-4 (44.7 kb) encodes two novel Rep_3 family proteins suggesting a co-integrate. Plasmid pD36-4 includes the *sul2* sulfonamide resistance gene, the *aphA1a* kanamycin/neomycin resistance gene in Tn*4352*::ISAba1 and a *mer* module in a hybrid Tn*501*/Tn*1696* transposon conferring resistance to mercuric ions. New examples of *dif* modules flanked by p*dif* sites (XerC-XerD binding sites) that are part of many *A*. *baumannii* plasmids were also identified in pD36-3 and pD36-4 which carry three and two *dif* modules, respectively. Homologs of three *dif* modules, the *sup* sulphate permease module in pD36-3, and of the *abkAB* toxin-antitoxin module and the orf module in pD36-4, were found in different contexts in diverse *Acinetobacter* plasmids, consistent with module mobility. A novel insertion sequence named ISAba32 found next to the p*dif* site in the *abkAB dif* module is related to members of the ISAjo2 group which also are associated with the p*dif* sites of *dif* modules. Plasmids found in D36 were also found in some other members of GC1 lineage 2.

## Introduction

*Acinetobacter baumannii* is a Gram negative opportunistic pathogen and a member of the ESKAPE group of bacteria that are the leading cause of difficult to treat nosocomial infections throughout the world [1]. *A*. *baumannii* has emerged as a global challenge mainly because of treatment failure due to its high levels of antibiotic resistance [2]. The majority of globally-distributed *A*. *baumannii* strains that are resistant to multiple antibiotics are members of the two main successful, globally-distributed clones, namely global clone 1 and global clone 2 or simply GC1 and GC2 [3–7].

We previously showed that the most recent common ancestor of GC1 arose around 1960 and subsequently around 1967 members of GC1 diverged into two phylogenetically distinct lineages [8]. In *A*. *baumannii*, unlike other Gram negative bacteria, resistance genes are often located in genomic resistance islands in the chromosome [5, 8, 9]. Strains belonging to the main GC1 lineage, lineage 1, all included either an AbaR-type resistance island or a remnant of it located in the chromosomal *comM* gene [8]. However, strains falling into lineage 2 contained either an intact *comM* gene or Tn*6022* or AbaR4 in *comM* [8]. AbaR4 is a 16.8 kb transposon consisting of Tn*6022*, a transposon related to the backbone transposon of the AbaR-type islands, interrupted by Tn*2006*, a transposon that carries the *oxa23* carbapenem resistance gene [10]. Recently, several studies have highlighted the significance of *A*. *baumannii* plasmids in introducing antibiotic resistance genes [11–14].

In an earlier study, we showed that D36 is a carbapenem resistant *A*. *baumannii* isolate that belongs to lineage 2 and ST81 (Institut Pasteur MLST scheme), which is a single locus variant (SLV) of the predominant GC1 sequence type, ST1 [8]. D36 was known to carry the AbaR4 resistance island in the *comM* gene [10]. However, D36 is resistant to other antibiotics including third generation cephalosporins, sulfamethoxazole, ciprofloxacin, gentamicin, kanamycin, neomycin and tobramycin [10, 15, 16]. The ISAba1-*ampC* structure accounts for its resistance to third generation cephalosporins [16] and mutations in *gyrA* and *parC* account for the fluoroquinolone resistance [8]. D36 also carries the *aadB* gene in a small plasmid called pRAY* explaining its resistance to kanamycin, tobramycin and gentamicin [15]. However, the context of the *aphA1a* and *sul2* genes that confer resistance to kanamycin and neomycin and to sulfamethoxazole, respectively, [17] had not been found.

In addition, the genome of D36 has been completely sequenced revealing three additional plasmids in this strain [17] but their properties were not described. Hence, here we sought to analyse the genetic structure of the plasmids carried by D36, compare them to other *Acinetobacter* plasmids and explore their distribution in other members of GC1, lineage 2 that are closely related to D36.

## Materials and methods

### Sequence analysis and bioinformatics

The strain D36 is an extensively antibiotic resistant strain recovered in 2008 at a Sydney hospital from a wound infection of a 27-year-old male who was a member of the armed forces.

The complete genome sequence of strain D36 was determined previously using a combination of Illumina HiSeq and PacBio technologies [17].

A range of bioinformatics tools was used to annotate the sequences of the three novel plasmids, pD36-1, pD36-3 and pD36-4 found in D36 (GenBank accession numbers CP012953, CP012955 and CP012956, respectively). The outputs of the automatic annotation program Prokka [18] were manually modified where further information such as assigned gene names was available. Antibiotic resistance genes were identified using ResFinder (https://cge.cbs.dtu.dk/services/ResFinder/) [19] and ISFinder (https://www-is.biotoul.fr/) was used to identify insertion sequences. Sequence repeats including iterons were found using Unipro UGENE v1.29.0 (http://ugene.net/). Pfam searches (http://pfam.xfam.org/) were also used to identify possible protein functions. The copy number of each plasmid was estimated by dividing the coverage of contigs containing plasmid sequences by the coverage of chromosomal contigs.

Gene Construction Kit (GCK 4.0.3) was used to visualize and manipulate the sequences studied. Figures were drawn to scale using GCK 4.0.3, SnapGene Viewer 4.1.7 and Adobe Illustrator CS6.

### Construction of pRAY*-Δ1

**Fig 1:**
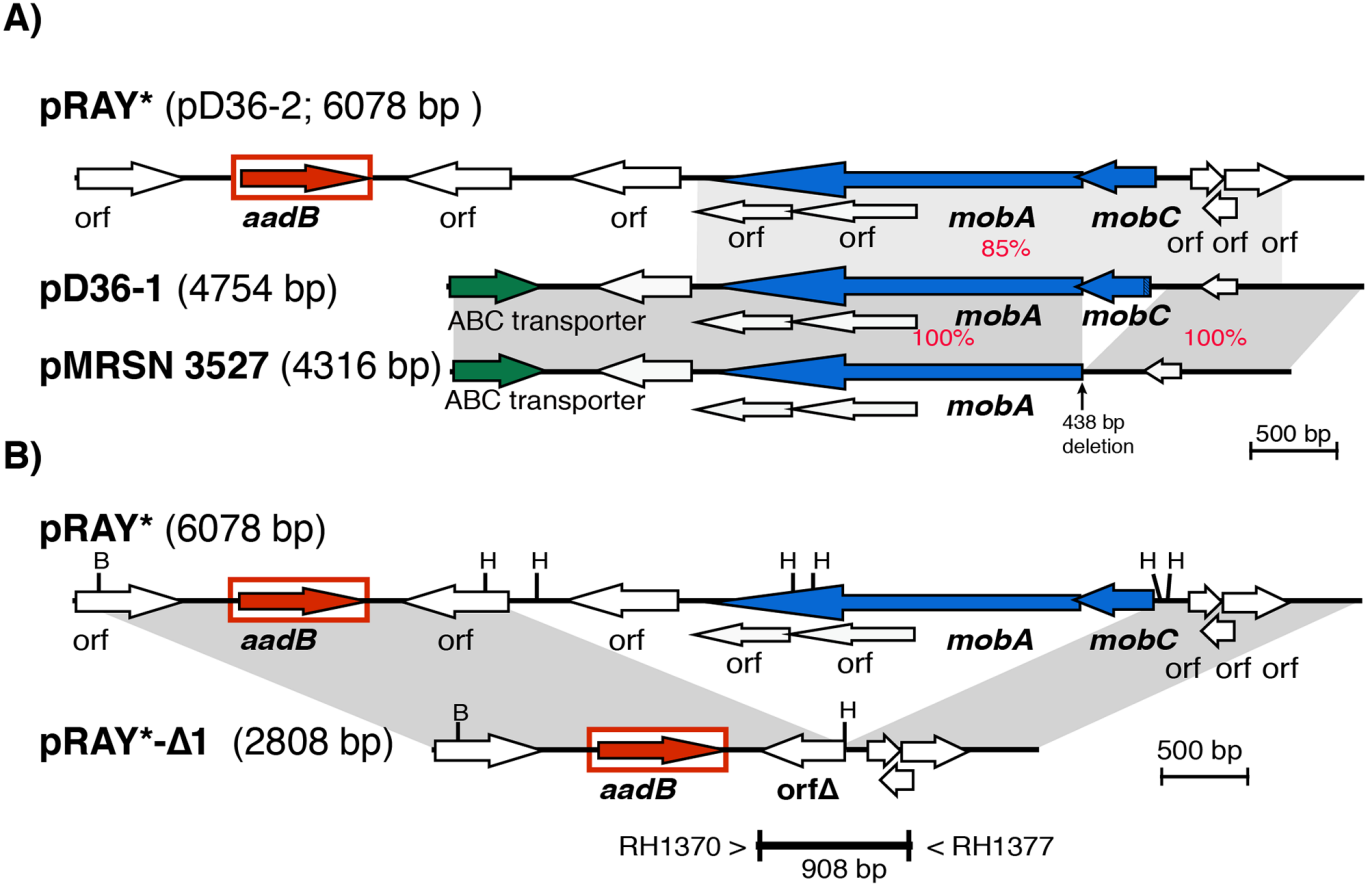
Linearized map of pD36-1 compared with pRAY* (pD36-2) and pMRSN 3527 (A) and comparison of pRAY* with pRAY*-Δ1 (B). Central horizontal lines indicate the plasmid backbones. Arrows represent the extent and orientation of genes and the gene cassette is boxed. The grey shadings indicate regions with significant identity with the % identities indicated in red. Scale bar is shown. Drawn to scale from GenBank accession numbers CP012954 (pRAY*), CP012953 (pD36-1), and JPHZ00000000 (pMRSN3527).

Plasmid DNA was isolated from D36, using the Wizard^®^ Plus SV Minipreps DNA Purification kit (Promega), and pRAY* was gel purified and digested with HindIII according to the manufacturer’s instructions. Approximately 50 ng of the HindIII digestion mix was then re-ligated using 1 μl of T4 DNA ligase (New England BioLabs, Ipswich, USA) and 2x Ligation buffer. The ligation mix was then used to transform the antibiotic susceptible *A*. *baumannii* strain AB307-0294 [7] by electroporation using 0.2 cm cuvettes and with the following parameters 2.5 kV, 25 μF and 200 Ω. Potential transformants were selected on L-agar supplemented with 20 mg/L kanamycin to select for pRAY* derivatives. To screen the deletion derivatives, the PCR primers RH1370 5'-CGTTATCGGATTTACTGCTTTAC-3' and RH1377 5'-CGTCAGCCCAATTACAGGTT-3', were designed to generate a product spanning all HindIII restriction sites present in pRAY* and used to amplify products with various length depending on the number of HindIII fragments present. The smallest PCR product obtained, representing the shortest pRAY* deletion derivative with only one HindIII site, was sequenced to confirm the deletion and the resulting corresponding plasmid, which lacked the *mobA* and *mobC* genes (Fig 1B) was named pRAY*-Δ1. Plasmid pRAY*-Δ1 was transformed into AB307-0294 (pA297-3) cells for mobilization assays. Transformants were selected on L-agar supplemented with 20 mg/L kanamycin and 100 mg/L sulphamethoxazole to select for both pA297-3 and pRAY*-Δ1. The resulting transformants were purified and the presence of both pA297-3 and pRAY*-Δ1 was confirmed by PCRs.

### Conjugation and mobilisation experiments

Conjugation was performed by mixing 100 μl of donor and recipient on an L-agar plate with no antibiotic selection. Following overnight incubation, cells were resuspended and diluted in 0.9% saline. Potential transconjugants were selected on Muller Hinton Agar (MHA) supplemented with nalidixic acid (25 mg/L) and sulfamethoxazole (100 mg/L), to select for pA297-3 transfer, and MHA agar containing nalidixic acid (25 mg/L), sulfamethoxazole (100 mg/L) and kanamycin (20 mg/L) to select for co-transfer of pA297-3 and pRAY* or pRAY*-Δ1. To examine whether the colonies selected with only sulfamethoxazole also carried pRAY* they were patched onto MHA containing kanamycin (20 mg/L). To ensure the colonies were transconjugants they were patched onto L-agar supplemented with 10 mg/L tetracycline, to which the recipient was sensitive and the donor resistant. Transconjugants were also screened by PCR to confirm plasmid transfer, pRAY* co-transfer and that the RepAci1 cryptic plasmid had not been co-transferred.

A rifampicin resistant mutant of the strain AB307-0294 [7], was generated by growing the cells overnight on L-agar supplemented with 100 mg/L rifampicin. Potential mutants were purified, confirmed phenotypically and one was named 307-0294^rif^. 307-0294^rif^ was then used as recipient and mated with AB307-0294 (pA297-3, pRAY*) as donor generated in the previous conjugation round. Transconjugants were selected on MHA containing 100 mg/L rifampicin and 100 mg/L sulfamethoxazole, to select for pA297-3 and on MHA with rifampicin, sulfamethoxazole and 20 mg/L kanamycin to select for pA297-3 and pRAY* co-transfer. Strain AB307-0294 (pA297-3, pRAY*-Δ1) was used as a donor and mated with AB307-0294^rif^ using the conditions and antibiotics specified above. The transfer and co-transfer frequencies were calculated using an average of values obtained from three replicates.

### Resistance to mercury

To examine whether D36 is resistant to mercury, 10 fresh colonies were patched onto L-agar supplemented with 25 μg/ml HgCl_2_ followed by overnight incubation at 37°C and visual inspection for the presence and absence of growth.

## Results

### Properties of the four plasmids carried by D36

The D36 genome consists of a chromosome (GenBank accession no. CP012952, 4063596 bp) and four plasmids named pD36-1 to pD36-4 and ranging in size from 4.7 to 47.2 kb [17]. The general features of these plasmids are summarised in Table 1. Two plasmids, pRAY* (pD36-2, 6 kb) described previously [15] and pD36-4 (47.4 kb) carry antibiotic resistance genes while the other two, pD36-1 (4.7 kb) and pD36-3 (7.2 kb) are cryptic [8]. The copy number of pD36-1, pRAY* and pD36-3 was estimated to be between 11–13 copies/cell while that of the largest plasmid, pD36-4, was 2–3 copies/cell.

**Table 1:** General features of plasmids found in D36. ^a^ Replication initiation protein. ^b^ Mobilisation protein. ^c^ pRAY* is pD36-2.

Plasmid	Size (bp)	Resistance genes	Copy number	Iteron	Rep^a^	Mob^b^	*dif* module	GenBank no.
pD36-1	4754	-	11–12	-	no Rep	MobA, MobC	-	CP012953
pRAY*^c^	6078	*aadB*	12–13	-	no Rep	MobA, MobC	-	CP012954
pD36-3	7239	-	11	+	Aci1	-	3	CP012955
pD36-4	47457	*sul2*, *aphA1a*, *mer*	2–3	- +	RepA1_,_ RepA2	MobC	2	CP012956

### pD36-1 and pRAY*

The smallest plasmid found in D36, the 4757 bp pD36-1, encodes only four proteins (Fig 1 and Table 1). The proteins include two potential mobilization proteins MobA (locus_id AN415_05003, protein_id ALJ89802.1), which is a putative relaxase (Pfam03432), and MobC (locus_id AN415_05004, protein_id ALJ89803.1), which is a relaxase accessory protein, as well as a putative transporter (AN415_05001, protein_id ALJ89800.1) and a hypothetical protein (AN415_05002). Notably, pD36-1 did not encode an identifiable replication initiation (Rep) protein indicating that it must be using an alternative mechanism to initiate replication. The closest relative for which a draft sequence is available, pMRSN3527 (GenBank accession no. JPHZ00000020) appears to be a deletion derivative (Fig 1A). Strain MRSN3527 also belongs to ST81 and lineage 2 GC1 [8]. Examination of its draft genome sequence (GenBank accession no. JPHZ00000000) revealed that a 438 bp portion of pD36-1 is missing.

The second plasmid, pD36-2 is pRAY*, which we previously recovered from D36 and sequenced [15], and it will be referred to as pRAY* hereafter. pRAY* (Fig 1) carries the *aadB* gene cassette which confers the gentamicin, kanamycin and tobramycin resistance [20] observed in D36. Comparison of pD36-1 and pRAY* revealed a 2929 bp region with 85% DNA identity spanning both *mobA* and *mobC* (Fig 1) indicating that the mobilization regions of pD36-1 and pRAY* are related. The origin of transfer has not been identified.

### pRAY* is mobilised by pA297-3

To examine the mobility of pRAY*, initially, the GC1 reference strain A297, which carries pRAY* and the large conjugative plasmid pA297-3 containing the *sul2* sulphonamide resistance gene [14] as well as the 8.7 kb cryptic RepAci1 plasmid predominantly found in GC1 isolates, was used as donor. When A297 was mated with AB307-0294, which is only resistant to nalidixic acid and does not contain any plasmids [7], the sulphonamide resistance on pA297-3 was transferred into AB307-0294 with a frequency of 1×10^−4^ (SD ±3.06×10^−5^) transconjugants/donor. Transconjugants selected for the presence of both plasmids (on MHA with sulfamethoxazole and kanamycin) arose at an average co-transfer frequency of 8×10^−5^ (SD ±5.95×10^−5^). 100 Su^R^ colonies from each of the three replicate conjugation experiments (300 in total) were patched onto MHA containing nalidixic acid, sulphamethoxazole and kanamycin and on average 81% (78/100, 81/100 and 84/100) of colonies grew. Similarly, 300 Su^R^Km^R^ transconjugants (Nx^R^) were purified and PCR assays confirmed that they contained both pA297-3 and pRAY*. Hence, all of the Su^R^Km^R^ transconjugants contained both pRAY* and pA297-3, indicating that pRAY* was co-transferred with pA297-3 at high frequency.

To examine whether *mobA* and *mobC* were required for mobilisation, a deletion derivative of pRAY*, pRAY*-Δ1 that lacks these genes (Fig 1B), was constructed. In the control conjugation, using AB307-0294 (pA297-3, pRAY*) as donor and AB307-0294^Rif^ as recipient, pRAY* co-transfer was detected in 88% of the 300 Su^R^Rif^R^ transconjugants colonies examined (85/100, 88/100 and 93/100 in three replicate experiments). In contrast, using AB307-0294 (pA297-3, pRAY*-Δ1) as the donor no Km^R^ transconjugants were detected after screening 300 Su^R^ colonies (100 from each replicate) for kanamycin resistance, indicating that pRAY*-Δ1 (Fig 1B) was not mobilised. Given that MobA and MobC of pRAY* are related to known relaxase/accessory combinations [15] this indicates a likely role for MobA and MobC in mobilisation of pRAY*. However, further work will be needed to establish whether both the relaxase (MobA) and the relaxase accessory protein (MobC) are required, as is usual in homologous systems.

### pD36-3—A RepAci1 plasmid

**Fig 2:**
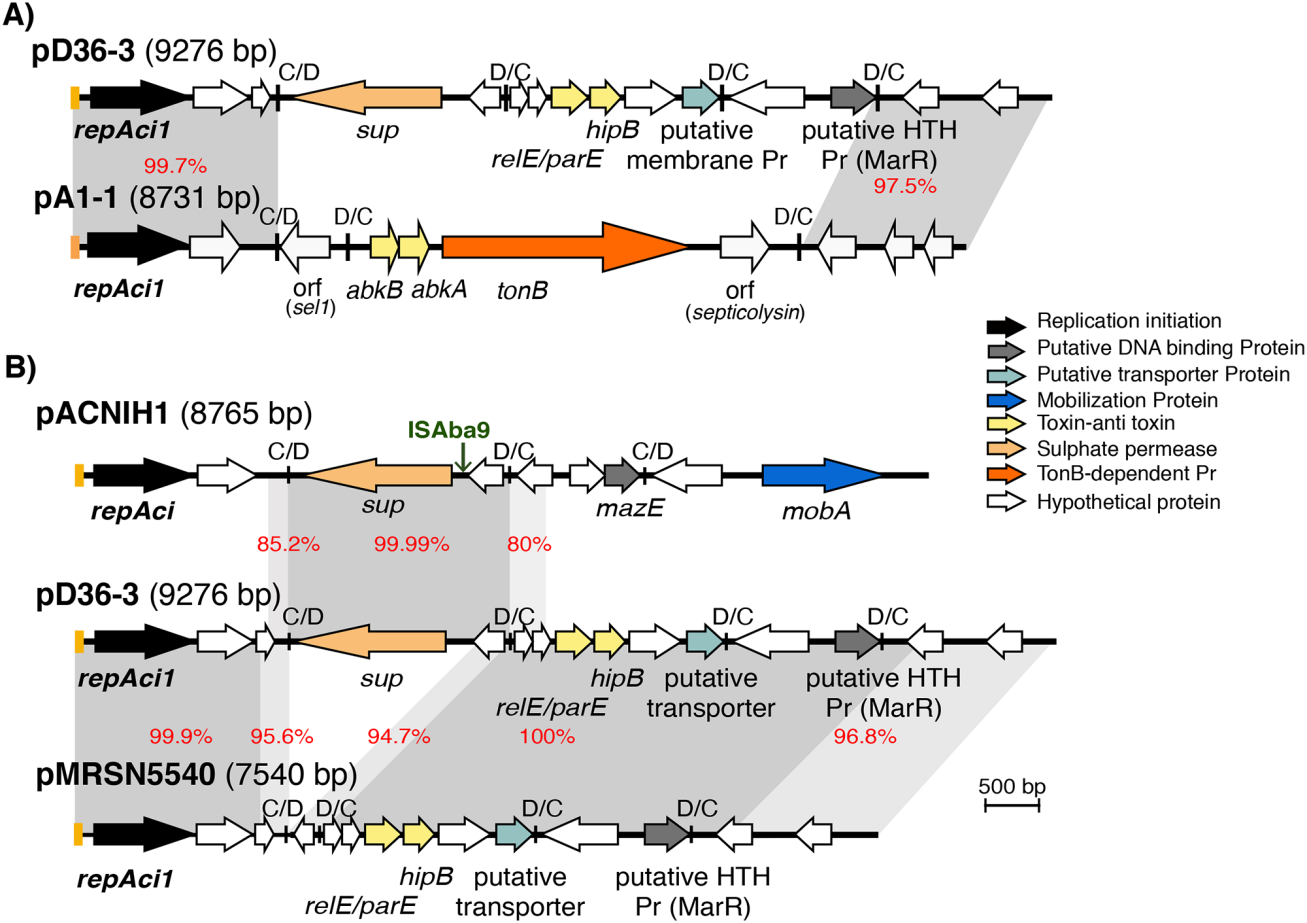
Linearized map of pD36-3 compared with pA1-1 (A) and pACNIH1 and pMRSN5540 (B). Central lines indicate the plasmid backbones with horizontal arrows indicating the extent and direction of genes/orfs. Genes/orfs are also colour coded according to the function they encode (shown below) and the key is shown on the right. The filled boxes coloured orange at the beginning of each line represent the iteron regions and small vertical bars indicated by C/D or D/C represent the two p*dif* orientations (XerC/D or XerD/C) binding sites. The scale bar is also shown. Drawn to scale from GenBank accession numbers CP012955 (pD36-3), CP010782 (pA1-1), CP026427 (pACNIH1) and LNCW01000064 (pMRSN5540).

The third plasmid, pD36-3 (9,276 bp), in strain D36 includes 15 orfs ranging in size from 144 bp to 1461 bp but carries no antibiotic resistance genes (Fig 2). It also encodes a Rep protein that is 100% identical to the previously identified RepAci1 replication initiation protein [21]. Examination of the region surrounding the *repAci1* gene indicated that it was preceded by a putative 88 bp iteron region consisting of four copies of the 22 bp repeat 5'-ATATGTCCACGTTTACCTTGCA-3' that is identical to the corresponding region in other RepAci1 plasmids such as pA1-1 (GenBank accession no. CP010782), which is an 8731 bp cryptic plasmid predominantly found in many members of global clone 1, lineage 1 including A297 (see above). In total a 1997 bp region on the left in Fig 2 (bases 1–1997 of pD36-3 including the *repAci1* gene), and 1652 bp on the right (bases 7624 to 9276) were 99.7% and 97.5% identical, respectively, to the corresponding regions in pA1-1 (Fig 2A).

Recently, a number of *A*. *baumannii* plasmids, including pA1-1, have been shown to include p*dif* sites, consisting of inversely oriented binding sites for the XerC and XerD recombinases separated by 6 bp [22–24]. Here, manual inspection of the pD36-3 sequence for consensus XerD and XerC binding sites indicated that the novel segment in pD36-3 contains four p*dif* sites, making three adjacent *dif* modules (Fig 2). Hence, pD36-3 is a novel variant of the group of plasmids encoding RepAci1 with a completely different array of *dif* modules.

#### The p*dif* modules of pD36-3

Generally, known *dif* modules in *Acinetobacter* species plasmids are flanked by inversely oriented p*dif* sites either with the XerD sites internal (C/D-D/C) or XerC sites internal (D/C-C/D) and the binding sites in an array of p*dif* modules should alternate [22]. However, in pD36-3 only the first *dif* module (on the left in Fig 2) followed this rule. This module was 2103 bp long, defined as the segment between the p*dif* sites on either side, and contained two orfs, one of which encodes a putative sulphate permease (Sup), which belongs to the large family of sulphate permeases (Pfam00916). The Sup encoded by pD36-3 was found to be 75% (365/485 aa) identical to the Sup encoded by the AbaR-type resistance islands predominantly found in the chromosomal *comM* gene of lineage 1 GC1 isolates [3]. The second orf (225 bp) found in this *sup* module encodes a hypothetical protein. The other two modules are flanked by two D/C sites suggesting that internal p*dif* sites have been lost. The middle *dif* module was 2010 bp in size and includes six orfs, two of which code for a putative *relE/parE-hipB* toxin-antitoxin pair (Fig 2). The last orf to the right of this module encodes a putative transcriptional regulator (Pfam13744) and the remaining orfs encode hypothetical proteins as Pfam searches could not assign any function to them. The third module was found to include 1462 bp between the two D/C p*dif* sites and contains two orfs, the first encoding a hypothetical protein and the second encoding a putative HTH-DNA binding protein (Pfam13730).

Searches of the GenBank non-redundant database revealed modules related to those found in pD36-3. The module containing the *sup* gene (*sup* module) (99.99% identity) was found in an unnamed 8765 bp *Acinetobacter* sp. plasmid (GenBank accession no. CP026427) (Fig 2B), here named pACNIH1 after its strain name, ACNIH1. This plasmid is cryptic and encodes a Rep protein identical to one of the two Reps (p2ABSDF0001) previously found [25] in p2ABSDF (GenBank accession no. CU468232). Additional modules related to (> 90% identity) the *sup* module of pD36-3 were also found in three unrelated plasmids including pALWED1.3 with 93% identity (GenBank accession no. KX426228) and pmZS (GenBank accession no. CP019144) and pZS-3 (GenBank accession no. CP019145), each with 95% identity to the module in pD36-3 indicating that this module belongs to a diverse family. Amongst these plasmids only pALWED1.3, which was recovered from permafrost and also carries chromium resistance determinants in a *dif* module, has been characterised [26].

The second module (2010 bp) that includes the *relE*/*parE*-*hipB* genes was not found in any other *Acinetobacter* plasmid though segments of this module ranging in size from 400 bp to 1.2 kb and with 85–95% DNA identities were found in several related entries (including plasmids and *Acinetobacter* spp. chromosomes). However, careful examination of the ends of these sequence alignments did not reveal the presence of additional p*dif* sites. The third *dif* module that encodes a putative transcriptional regulator was found to be completely novel as it was not found in any other entry.

However, searches of the WGS database revealed a set of related *A*. *baumannii* plasmids that carried two of the modules found in pD36-3 (represented by pMRSN5540 in Fig 2B, GenBank no. LNCW01000064). Our analysis indicated that the strain MRSN5540 is a member of GC1, lineage 1 but it belongs to ST94, which is a double locus (*fusA* and *gltA*) variant of ST1. In pMRSN5540, two short regions with ~95% sequence identity, compared to their corresponding regions on the left-end of the *sup* module and spanning the internal orf of the *sup* module on the right, were found next to each other (Fig 2B). Consequently, pMRSN5540 lacked the *sup* gene due to an internal deletion starting from 4 bp inside the *dif* module on the left and extending 1755 bp to the 3'-end of the internal orf on the right (Fig 2B). The cause of this deletion was unclear as careful inspection of this module did not reveal additional p*dif* sites. The sequence of this region could not be confirmed as MRSN5540 was not available to be examined further.

### pD36-4 carrying resistance genes and *dif* modules

**Fig 3:**
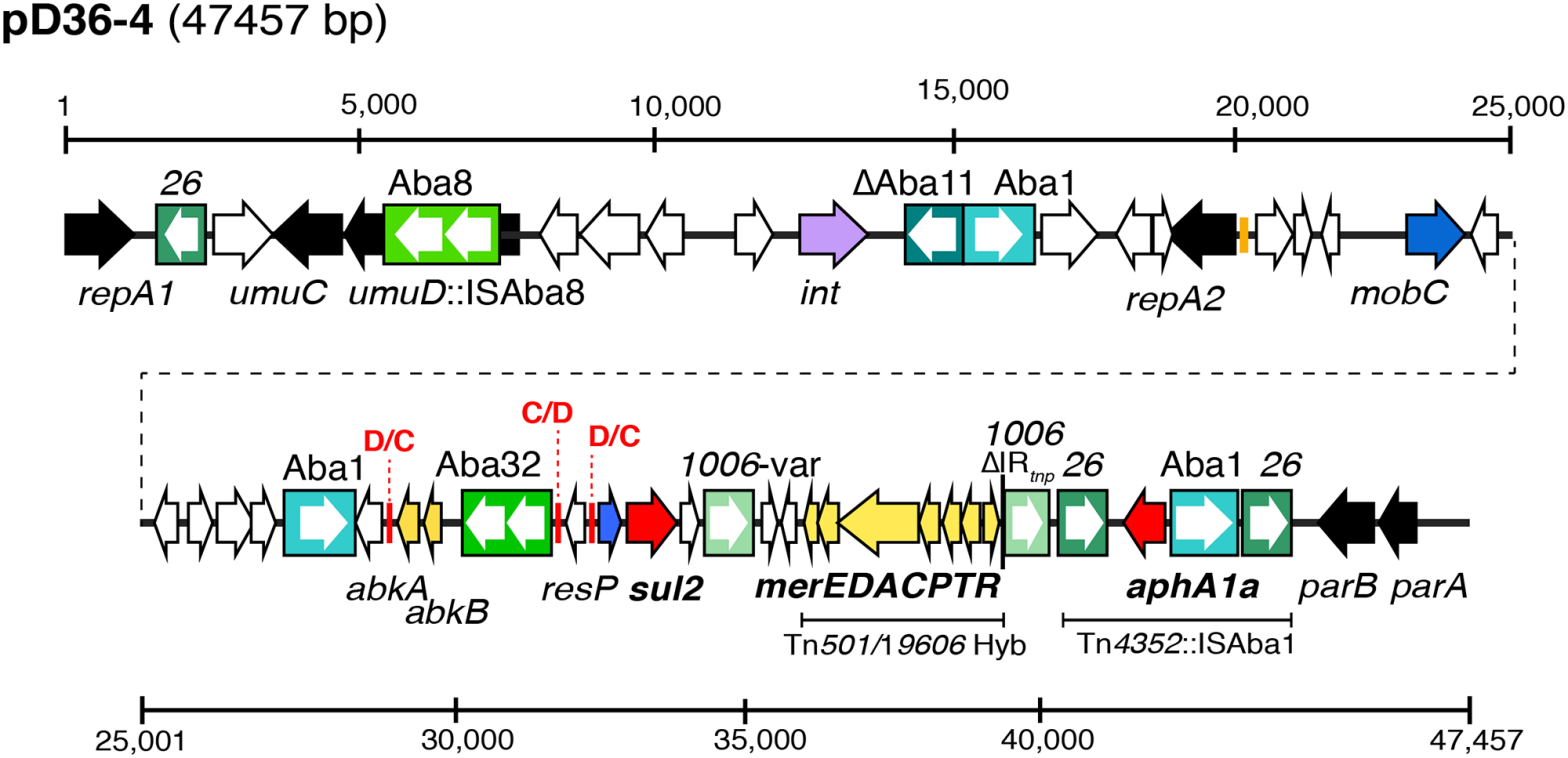
Linearized map of pD36-4. The thick horizontal line represents the plasmid backbone and arrows indicate the extent and orientation of genes/orfs. Filled boxes coloured different shades of green indicate insertion sequences with the white arrows inside indicating the direction of the transposase genes. Small vertical red bars indicated by C/D or D/C represent p*dif* binding sites. The vertical bar coloured orange upstream of *repA2* indicates the iteron region. The scale bar is also shown. Drawn to scale from GenBank accession no. CP012956.

The largest plasmid found in D36 was pD36-4 (GenBank accession no. CP012956), which is 47457 bp in length [17] and includes 57 orfs ranging in size from 138–1686 bp. Two novel Rep proteins (Fig 3), which belong to the Rep_3 family of replication initiation proteins (Pfam01051), were encoded by pD36-4 and were named RepA1 (locus_taq AN415_8001, protein_id ALJ89824.1) and RepA2 (locus_taq AN415_8021, protein_id ALJ89842.1), respectively (Fig 3). Compared to known RepA proteins reported in the published typing scheme [25], the closest match to RepA1 was a RepA protein encoded by p3ABAYE (290/392 aa) (protein_id CAM84695.1; locus_id p3ABAYE0002 in GenBank accession no. CU459140) that is 74% identical, and RepA2 was 57% identical (176/307 aa) to the RepA encoded by pAB1, which is a 13 kb (GenBank accession no. CP000522) plasmid found in ATCC 17978. RepA2 is also 91% identical to one of the RepA proteins found subsequently in the unrelated plasmid pAb242_25 (GenBank accession no. KY984047), which was sequenced recently [27, 28]. Examination of the regions surrounding the *repA* genes showed that *repA2* is preceded by three copies of a putative 19 bp iteron sequence 5'-AGGTGGGAAAACGTAGACT-3' (orange vertical line in Fig 3) but iterons were not found in the region upstream of *repA1*.

The *aphA1a* kanamycin, neomycin resistance gene and *sul2* sulfonamide resistance gene are both found in pD36-4 [17]. The *aphA1a* gene is located within Tn*4352* [29] but the central segment was interrupted by an ISAba1 upstream of *aphA1a* in the correct orientation to enhance transcription. The *sul2* sulphonamide resistance gene found in pD36-4 is derived from GI*sul2* [30] and is located downstream of a *resP* gene encoding a putative resolvase (Fig 3). It was found that pD36-4 also carries a hybrid Tn*501*/Tn*1696* hybrid mercury resistance module that extends to within the 38 bp IR usually found at the boundary of these transposons. The *A*. *baumannii* isolate D36 was found to be resistant to mercuric ions and given that no other gene(s) responsible for mercury resistance were found in other plasmids or in the chromosome, the Tn*501*/Tn*1696* module confers the observed mercury resistance of D36. In addition to the three IS associated with Tn*4352*::ISAba1, the backbone of pD36-4 included eight complete or partial IS (Fig 3).

#### The p*dif* modules of pD36-4

**Fig 4:**
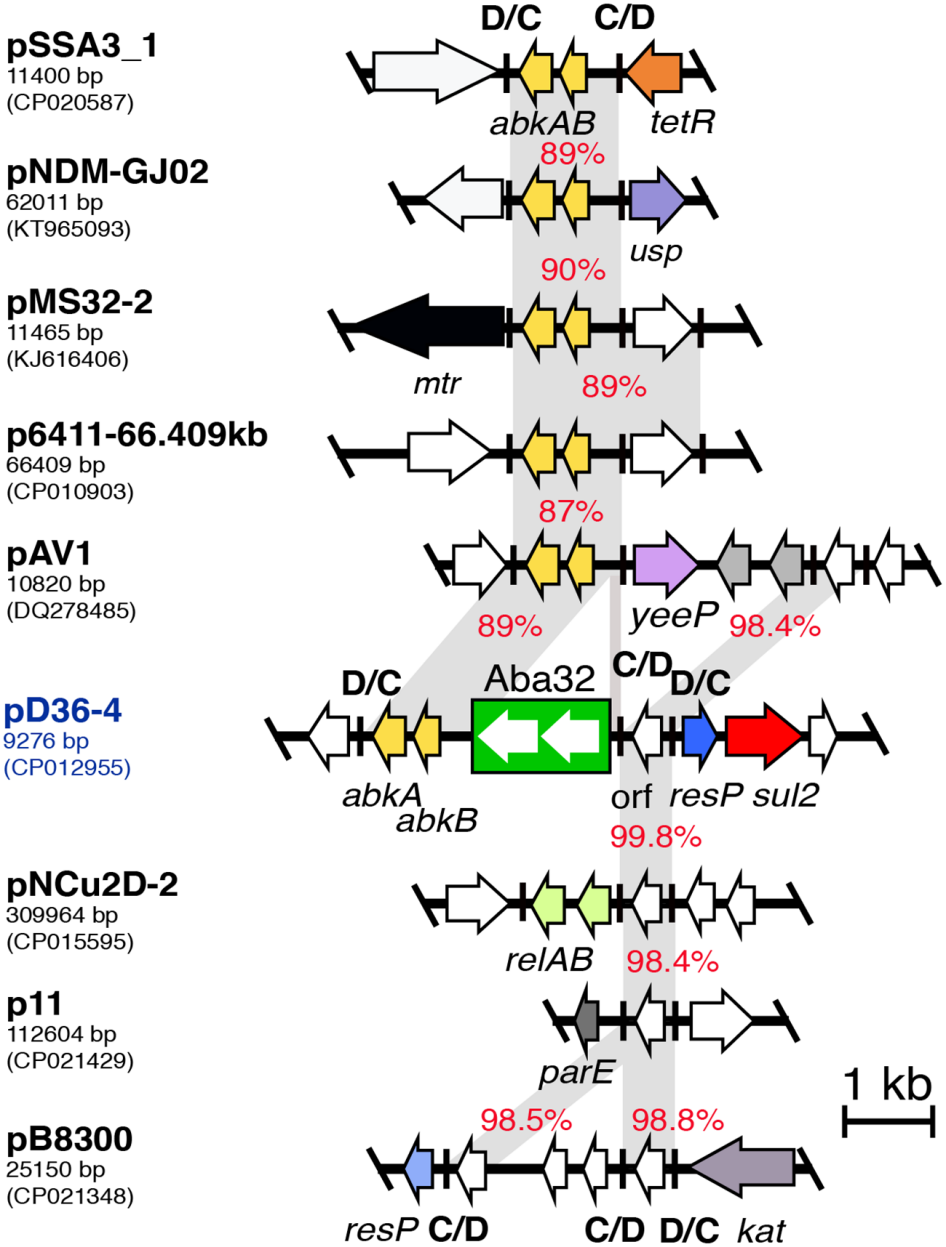
Comparison of *dif* modules found in pD36-4 to related modules from diverse *Acinetobacter* plasmids. Only 1–2 kb of the surrounding region of each *dif* module is shown. Central black lines represent the backbone of plasmids and vertical lines indicate p*dif* sites (marked C/D or D/C to indicate their orientation). Horizontal arrows indicate the extent and orientation of orfs. Open reading frames encoding hypothetical proteins are white. Segments with significant identities (>90%) are connected using a shade of grey with the numbers indicating identities. In pNDM-GJ02 the *usp* gene encodes a universal stress protein and *mtr*, in pMS32-2, encodes a DNA methyltransferase. The figure is drawn to scale using the sequence retrieved from the GenBank accession numbers indicated for each plasmid. Scale bar is shown.

Examination of the pD36-4 sequence revealed that it carries three p*dif* sites making two *dif* modules, a C module (bounded by D/C and C/D sites) adjacent to a D module (bounded by C/D and D/C sites). The D module was only 572 bp in size and contains a single orf (Fig 3). The fragment contained in the larger *dif* module is 2310 bp long and includes two orfs encoding the AbkA (pfam14384) and AbkB (pfam04365) toxin-antitoxin proteins [31] as well as a copy of a novel insertion sequence, ISAba32 (Fig 3). Modules related to the uninterrupted *abkAB* module (822 bp), were found in different genetic contexts in five *Acinetobacter* plasmids (Fig 4). Though none of the *dif* modules are identical, they are each flanked by different sequences indicating that these modules can move between plasmids.

Searches of the GenBank using the smaller *dif* module as query also revealed six entries with a related module including the four *Acinetobacter* plasmids shown in Fig 4, and two *Acinetobacter* chromosomes (GenBank accession no. CP012035 and CP018260). Again, the flanking sequences are different as expected for a mobile element.

#### ISAba32 a novel IS found in pD36-4

ISAba32 was found first in pD36-4 and its sequence deposited in the ISFinder database (https://www-is.biotoul.fr/scripts/ficheIS.php?name=ISAba32). ISAba32 is 1482 bp long and has 26 bp terminal inverted repeats. It was inserted 5 bp away from the XerC binding site of a p*dif* site and has created a 5 bp direct duplication on insertion. We found a number of unnamed ISs with sequence identity to ISAba32 of 89–98% in at least eight entries in GenBank non-redundant database. These entries included eight *Acinetobacter* plasmids (GenBank accession numbers CP020587, KT965093, KJ616406, CP010903, DQ278485, CP015595, CP021429 and CP021348) as well as the chromosome of the *Acinetobacter radioresistens* strain DSSKY-A-001 (GenBank Accession no. CP027365). In each of these sequences, the IS was found to be located precisely 5 bp away from a XerC binding site and was always in the same orientation. ISAba32 is also 73.3% identical to ISAjo2, which is found in the same position [22] thus both the ISAba32 group and the ISAjo2 group clearly target *dif* sites.

### Distribution of D36 plasmids in members of GC1 lineage 2

The draft genome sequences of seven strains, which were previously found to belong to GC1 lineage 2 [8], were examined here for the presence of the plasmids found in D36. A deletion variant of pD36-1 was found in *A*. *baumannii* MRSN 3527 (Fig 1) while none of the other strains contained pD36-1 or a variant (Table 2). Plasmid pRAY*, which is widespread [15], was found in all but TG19582 and OIFC074. In addition, even though the genome sequences of all of the strains examined here were still in the WGS database, our searches of the contigs in draft genomes led to the conclusion that all three ST81 strains, 6013150, 6013113 and MRSN 3527, are likely to contain both pD36-3 and pD36-4 or a variant of them (Table 2). This is consistent with the ST81 isolates being part of a clade within lineage 2 of GC2 as shown previously [8].

**Table 2:** Distribution of plasmids found in D36 in other members of GC1, lineage2.

Isolate	Date	Country	Source	pD36-1	pD36-2 (pRAY*)	pD36-3	pD36-4	*aphA*^a^	*mer*	*sul2*	ST_IP_	GenBank no.
TG19582	nk^b^	nk	nk	-	-	-	-	-	-	+	1	AMIV00000000
Naval-21	2006	US	Wound	-	+	-	-	1b	-	+	19	AMSY00000000
OIFC074	2003	US	nk	-	-	-	-	1b	-	+	19	AMDE01000000
D36	2008	Australia	Wound	+	+	+	+	1a	+	+	81	CP012952
6013150	2007	UK	Skin	-	+	+^c^	+	1a	+	+	81	ACYQ00000000
6013113	2007	UK	Skin	-	+	+^c^	+	1a	+	+	81	ACYR00000000
MRSN 3527	2011	US	Wound	+^d^	+	+^e^	+^f^	6	+	+	81	JPHZ00000000

^a^ *aphA1a*, *aphA1b* or *aphA6*.

^b^ not known.

^c^ Includes indels ~ 0.3 kb and 1.3 kb.

^d^ 438 bp missing.

^e^ Containing only 2 fragments (0.7 and 2.2 kb) of pD36-3.

^f^ Missing the Tn*4352*::ISAba1 structure likely due to an IS*26*-mediated deletion event.

## Discussion

Our previous study indicated that the two phylogenetically distinct lineages of *A*. *baumannii* GC1 acquired resistance to antibiotics via different routes [8]. However, how members of lineage 2 became resistant to several antibiotics is not well understood. Here, the plasmids found in D36, an extensively antibiotic resistant member of lineage 2 [17], were studied. The largest plasmid, pD36-4, contains two antibiotic resistance genes, *aphA1a* in Tn*4352* interrupted by ISAba1 and *sul2* in a fragment from GI*sul2*. It also included a Tn*501*/Tn*1696* hybrid *mer* region responsible for resistance to mercuric ions (Fig 3). Hence, two plasmids, pRAY* and pD36-4 carry resistance genes, complementing the chromosomal resistance determinants. Plasmids pRAY* (pD36-2), pD36-3 and pD36-4 were found to be present in all ST81 isolates regardless of the country of origin (Table 2). Hence, it appears that the ST81 group represent a distinct multiply resistant clade of lineage 2 isolates and this is consistent with the recombination-free phlyogeny generated from the chromosomes of three members of this group [8].

Plasmid pRAY* was mobilised by pA297-3, a large conjugative plasmid, and this is likely to be an important route for the spread of the *aadB* gentamicin, tobramycin and kanamycin resistance gene. We previously reported that the N-terminal end of the putative MobA encoded by pRAY* is related to MbeA of ColE1, and is therefore classified as a member of the MOB_P5_ (formerly MOB_HEN_ [32]) group of the MobA superfamily [15]. Here, we demonstrated that pRAY* was mobilised by pA297-3 only if *mobA* and *mobC* genes were present. Plasmid pA297 [14] is of a type seen to date only in *Acineobacter baumannii*. The small cryptic plasmid pD36-1 includes a region spanning the *mobA* and *mobC* genes related to the corresponding region in pRAY*. The predicted MobA and MobC proteins of pD36-1 and pRAY* were 77% and 94% identical, respectively, and it is possible that pD36-1 can also be mobilised by pA297-3 and related conjugative plasmids. A small plasmid pALWED1.8, which was found in an *A*. *lwoffii* recovered from permafrost and carries the *aadA27* streptomycin/spectinomycin resistance gene also carries a mobilisation region related to that of pRAY* [33]. In fact, mobilisation of pALWED1.8 was demonstrated [33] but the sequence of the conjugative plasmid used is not available precluding comparison to pA297-3. Further work is needed to examine these questions.

Neither pD36-1 nor pRAY* included an identifiable *rep* gene suggesting an alternative replication mechanism. Indeed, it is possible that like ColE1 [34] pRAY* uses an antisense RNA mechanism to initiate its replication. Notably, pRAY*-Δ1, which was constructed here for mobilisation assays, is only 2808 bp in size (2217 bp + *aadB* cassette) but is still able to replicate indicating that it contains all of the sequences required for initiation of replication. However, further deletions will be required to determine the exact sequences essential for replication initiation.

D36, the GC1 lineage 2 *A*. *baumannii* isolate studied here, carried two plasmids, pD36-3 and pD36-4, that contained *dif* modules. One of them, pD36-3 is a RepAci1 plasmid. RepAci1, is seen in the 8.7 kb pA1-1 plasmid (Fig 2A) frequently found in members of GC1 [11, 35, 36]. However, pD36-3 has a novel structure due to a different array of *dif* modules. Though we did not find identical modules in GenBank, close relatives of some of the pD36-3 and pD36-4 *dif* modules were found in different contexts in other *A*. *baumannii* plasmids providing evidence that these modules are mobile and can spread amongst *Acinetobacter* plasmids. The divergence in the sequences of these sets of related *dif* modules speaks of a long history for this type of mobile element and this is supported by the finding of one of them in a bacterium recovered from permafrost [26]. Clearly, *dif* modules are playing a significant part in the evolution of *Acinetobacter* plasmids and, though an initial insight into how they move has been provided recently [28], this warrants further investigation. A further insight into our understanding of the properties of *dif* modules comes from our analysis of the *abkAB dif* module in pD36-4 encoding the AbkAB toxin-antitoxin system. This module is also related (90% identical) to one end of the large *abkAB*-*tonB*-septicolysin module in pA1-1 (see Fig 2A). The similarity extends from the left hand p*dif* sites to the inner edge of the p*dif* site at the other end which is missing from the module in pA1-1. Hence, as we suggested previously [22], the p*dif* site at the right-hand end of the *abkAB* module has been lost, explaining the fact that the larger module in pA1-1 is flanked by directly-oriented p*dif* sites.

ISAba32, a novel IS that was found in pD36-4, along with a number of related but unnamed IS found in GenBank, was located 5 bp away from the XerC binding site of a p*dif* site. Recently, another IS, ISAjo2 and related several IS have also been reported to be inserted 5 bp away from the XerC binding site within a p*dif* site of several *Acinetobacter* plasmids [22]. Both ISAba32 and ISAjo2 are 1482 bp in size and are yet to be classified into a named IS family. The ISAba32 and ISAjo2 DNA sequences are 73.3% identical, and the ISAba32 encoded transposase is 72% identical to that of ISAjo2. Hence, it appears that the variants of ISAjo2 and ISAba32 can specifically target *dif* modules and insert 5 bp away from a XerC binding site. The targeting mechanism is unknown and warrants further investigation.

## Conclusions

The three novel plasmids found in an *A*. *baumannii* strain D36 increase our knowledge of the features and structures found in the unique pantheon of plasmids found in this species and more broadly in the *Acinetobacter* genus. Two plasmids contributed to antibiotic resistance and pRAY*, the fourth plasmid in D36, is mobilizable at high frequency by a conjugative plasmid of a type found to date only in *A*. *baumannii*. Some of the *dif* modules found in two of the plasmids were found elsewhere adding to the increasing evidence that *dif* modules are mobile elements, and an IS in one of them clearly represents a so far unrecognised family of IS that target *dif* modules.
